# Student public commitment in a school-based diabetes prevention project: impact on physical health and health behavior

**DOI:** 10.1186/1471-2458-11-711

**Published:** 2011-09-20

**Authors:** Lynn L DeBar, Margaret Schneider, Kimberly L Drews, Eileen G Ford, Diane D Stadler, Esther L Moe, Mamie White, Arthur E Hernandez, Sara Solomon, Ann Jessup, Elizabeth M Venditti

**Affiliations:** 1 Kaiser Permanente Center for Health Research, 3800 North Interstate Ave, Portland, OR 97227, USA; 2 Department of Planning, Policy, and Design, University of California at Irvine, 258 Social Ecology I, Irvine, CA 92697, USA; 3 The George Washington University Biostatistics Center, 6110 Executive Blvd, Suite 750, Rockville, MD 20852, USA; 4 The Children's Hospital of Philadelphia, Department of Pediatrics, 3535 Market Street, Suite 1572, Philadelphia, PA 19104, USA; 5 Division of Health Promotion and Sports Medicine, Oregon Health & Science University, 3181 SW Sam Jackson Park Road, CR110, Portland, OR 97239, USA; 6 Children's Nutrition Research Center, Baylor College of Medicine, 1100 Bates Street #2033, Houston, TX 77030, USA; 7 College of Education, Texas A&M University, Corpus Christi, 6300 Ocean Drive, Unit 5818, Corpus Christi, TX 78412, USA; 8 City of Philadelphia Department of Public Health, 1101 Market Street, Floor 9, Philadelphia, PA 19102, USA; 9 School of Nursing, The University of North Carolina at Chapel Hill, Carrington Hall, CB #7460, Chapel Hill, NC 27599-7460, USA; 10 University of Pittsburgh, Western Psychiatric Institute and Clinic, 3811 O'Hara St., Pittsburgh, PA 15213, USA

## Abstract

**Background:**

As concern about youth obesity continues to mount, there is increasing consideration of widespread policy changes to support improved nutritional and enhanced physical activity offerings in schools. A critical element in the success of such programs may be to involve students as spokespeople for the program. Making such a public commitment to healthy lifestyle program targets (improved nutrition and enhanced physical activity) may potentiate healthy behavior changes among such students and provide a model for their peers. This paper examines whether student's "public commitment"--voluntary participation as a peer communicator or in student-generated media opportunities--in a school-based intervention to prevent diabetes and reduce obesity predicted improved study outcomes including reduced obesity and improved health behaviors.

**Methods:**

Secondary analysis of data from a 3-year randomized controlled trial conducted in 42 middle schools examining the impact of a multi-component school-based program on body mass index (BMI) and student health behaviors. A total of 4603 students were assessed at the beginning of sixth grade and the end of eighth grade. Process evaluation data were collected throughout the course of the intervention. All analyses were adjusted for students' baseline values. For this paper, the students in the schools randomized to receive the intervention were further divided into two groups: those who participated in public commitment activities and those who did not. Students from comparable schools randomized to the assessment condition constituted the control group.

**Results:**

We found a lower percentage of obesity (greater than or equal to the 95^th ^percentile for BMI) at the end of the study among the group participating in public commitment activities compared to the control group (21.5% vs. 26.6%, p = 0.02). The difference in obesity rates at the end of the study was even greater among the subgroup of students who were overweight or obese at baseline; 44.6% for the "public commitment" group, versus 53.2% for the control group (p = 0.01). There was no difference in obesity rates between the group not participating in public commitment activities and the control group (26.4% vs. 26.6%).

**Conclusions:**

Participating in public commitment activities during the HEALTHY study may have potentiated the changes promoted by the behavioral, nutrition, and physical activity intervention components.

**Trial Registration:**

ClinicalTrials.gov number, NCT00458029

## Background

Epidemic rates of childhood and adolescent obesity represent a serious public health concern. Obesity prevalence among youth remains at historically high levels with 16.0% of 6 to 19 year olds overweight and 18.7% obese [1]. Higher rates are reported among economically disadvantaged and minority youth [1-3]. The health and economic burden of such morbidity is substantial [4], including, most seriously, risk for type 2 diabetes [4,5]; hence interventions aimed at preventing and controlling childhood obesity are increasingly important. Because schools are uniquely positioned to promote healthful eating and physical activity, school-based interventions provide an unparalleled opportunity to reach many of the highest risk youth [6,7]. Many programs have been designed and launched to address these targets [7-10], yet outcomes have been disappointing [7,11,12]. Finding ways to increase students' commitment to program goals, particularly strategies that leverage the social environment and peer influence of young adolescents, may contribute to better outcomes and more sustained and generalized effects.

Strong theoretical and empirical evidence supports the supposition that individuals who make a public commitment to a behavioral goal exhibit greater effort and persistence. This supposition, investigated in both laboratory and field settings, is grounded in work demonstrating that public commitment to an attitudinal position enhances resistance to persuasion [13-15] as well as in the research on strategic self-presentation [16]. Salancik [17] posited that individuals are strongly motivated to appear rational and consistent, and that "one of the simplest ways to commit yourself to a course of action is to go around telling all of your friends that you are definitely going to do something." College students randomly assigned to make a public (vs. private) commitment to a goal demonstrated stronger commitment [18], greater goal-consistent behavior [19], more effort expended to reach the goal [20], and greater progress toward the goal [21]. These laboratory-based studies show that individuals who publicly commit to a performance goal are more likely to enact behaviors that move them toward their goal compared to those who make a private statement of intentions. In addition, Wilson and colleagues [22] suggest that there is a "carry-over effect" whereby an individual's self presentation strongly influences their private self-appraisal and, in turn, subsequent behavior. These authors suggest that this effect is due to cognitive dissonance [23,24] (altering self-concept to match how one publicly presents oneself to reduce dissonance when one's private self-concept and public self-presentation are discrepant) and biased scanning [25] (in which beliefs congruent with one's actions are most salient and primed).

Outside the controlled laboratory environment, public commitment to a behavioral goal has also been shown to influence health-related behaviors. More than fifty years ago, classic studies found those who publicly agreed to change their dietary habits were more likely to do so and to sustain these changes than were those who made no such commitment [26,27]. Such decisional processes were also shown in studies with youth [28]. More recently, making a public commitment has been shown to increase adherence to medicinal prescriptions [29], and has been incorporated into school-based health-behavior interventions, such as smoking prevention programs [30-32] and dietary interventions [33]. Most pertinent to the current analysis, Birnbaum and colleagues [33] reported that in a middle school-based nutrition intervention, peer leaders were the only student participants to demonstrate consistent and significant improvements in fruit-and-vegetable consumption and lower-fat food choices. The authors suggest that peer leaders may have changed their behavior to reduce cognitive dissonance or to "walk the talk", given that they delivered eating-related messages to classmates and may have felt pressure (conscious or not) to make their behavior conform to those messages. As noted by Wilson and colleagues [22], studies examining the role of commitment in producing behavior change suggest that "individuals who ***freely ***choose to commit themselves publicly to a particular identity ('I eat healthy') and a course of action should be more likely to do so than individuals who only hold such beliefs privately".

For the current report, students in the HEALTHY study who visibly and voluntarily aligned themselves with HEALTHY-sponsored activities can be said to have made **a *public commitment ***to the study's behavioral goals (being more active and consuming nutritious foods and beverages). The ways that students demonstrated public commitment included a variety of activities, such as: assisting with classroom activities, helping with grade and/or school-wide events such as taste tests and cafeteria learning events, and inviting peers' participation; making public-address announcements; wearing study-related t-shirts identifying them as peer leaders; and being featured in print media posted throughout the school. Each demonstration of public commitment to the study's behavioral goals manifested the student's adoption of his or her peer leadership role. Here we examine whether making a public commitment to the HEALTHY program over the course of the intervention improved study outcomes including reduced obesity and improved health behaviors.

## Methods

### Study design

HEALTHY was a multi-center primary prevention trial designed to reduce risk factors for type 2 diabetes in adolescents. The overall trial design, and the communications component specifically, are described in detail elsewhere [34,35]. HEALTHY was a cluster-design trial [36] where schools were the unit of randomization and intervention. The primary outcome of the study was body mass index (BMI) percentile. Data were collected at both the school and the student level, but only from those individuals providing assent and parental informed consent. A cohort of 4603 sixth grade students who were enrolled and for whom data were available at baseline and at the end of their eighth grade year were included in the primary outcome analyses. Overall findings at the end of eighth grade included greater reductions in the intervention schools on BMI z-score, percentage of students with waist circumference at or above the 90^th ^percentile, fasting insulin levels (P = 0.04 for all comparisons), and prevalence of obesity (P = 0.05)[37].

The HEALTHY intervention, delivered over five semesters (Spring 2007, Fall 2007, Spring 2008, Fall 2008, Spring 2009) comprised four components: 1) nutrition; 2) physical education; 3) behavior; and 4) communications. The four intervention components were integrated by a series of themes targeting specific behaviors and building on each other (i.e., consuming water versus added sugar beverages; increasing physical activity and reducing sedentary behavior; consuming high-quality and reducing low-quality food; achieving energy balance; and making healthy choices for life). These themes are described elsewhere [34,38-40]. Theme-based communications campaigns [34] integrated and supported each aspect of the HEALTHY intervention and used core elements, such as branding, posters, banners, visual and verbal messaging; student events and student-generated media; distribution of thematic branded items, such as water bottles and pedometers; and involvement of student peer communicators (SPC, described in more detail below). Communications intervention strategies, including public commitment opportunities for students, were intended to strengthen the impact of all HEALTHY intervention components.

### Study Participants

Forty-two schools participated in the study (21 matched pairs randomized to intervention and control conditions), representing a broad geographic distribution (California, North Carolina, Oregon, Pennsylvania, and Texas). Major inclusion criteria for schools were at least 50% of children eligible for federally subsidized, free, or reduced-priced meals and/or at least 50% of students whose ethnicity was Black or Hispanic. To be eligible for the study cohort, students had to: be enrolled in 6^th ^grade in Fall 2006; provide data to determine BMI percentile at baseline; and have no conditions that would preclude active participation in physical education classes. Fourteen students who had a previous diagnosis of diabetes at baseline and one student who was identified as meeting the criteria for diabetes at baseline were excluded. Almost two-thirds of students at the targeted schools agreed to participate in the data collection (58.9%) and there was little difference between those who consented and assented and those who did not with respect to mean (± SD) BMI (the weight in kilograms divided by the square of the height in meters) (22.6 ± 8.7 and 21.8 ± 5.3, respectively), mean age (11.3 ± 0.6 and 11.3 ± 0.7 years), race or ethnic group (70.5% and 72.9% black or Hispanic), or sex (47.7% and 53.0% boys) [35]. All children in the intervention schools and grades received the intervention. The study was approved by each participating university's Institutional Review Board (IRB). Recruitment procedures, described in detail elsewhere [41], were identical for intervention and control schools.

### Public Commitment Activities: Student Peer Communicators

Students in the cohort grade known as student peer communicators (SPC) endorsed and promoted study activities to their peers and provided informal feedback to HEALTHY study staff. Participation was voluntary and the number of SPCs at each school depended on school size and other local considerations. SPCs were selected through a combination of self- and peer-nomination. The SPC was seen as a potential "influencer" or one who was able to promote key study messages in a meaningful way to peers. SPCs "connected" students in the cohort grade to the HEALTHY intervention through the dissemination of study messages, facilitation of classroom-based, cohort- and school-wide activities, and informal communication with their peers about study activities [34]. All SPCs attended a one-hour initial training that was standardized by supplying study staff with a centrally-designed PowerPoint presentation. The initial training outlined the required tasks, skills, and procedures, and included the voluntary recitation of the following pledge:

"I promise to be a HEALTHY leader in my school. I will learn about being HEALTHY and share what I learn with my friends, my school, my family and my community. I will be positive and encouraging. I will set an example to the best of my ability by living well in every way. I am HEALTHY!

This initial training was followed in subsequent weeks by supplemental 30-minute trainings specific to each intervention activity in which each SPC participated. SPCs received training for each activity they selected. The study protocol dictated that students would be required to spend no more than one hour per week of time outside class, however, all SPCs were expected to be active in study-related events throughout the duration of their participation. Table 1 describes specific activities and shows the frequency with which students served in these roles.

**Table 1:** Summary of Student Public Commitment Activity

	% (N)		
			
Type of Activity			
SPC only	13.8% (318)		
SGM only	13.5% (312)		
SPC/SGM	8.9% (205)		
none	63.8% (1472)		
Semesters of Participation			
1	12.8% (295)		
2	12.7% (294)		
3	6.9% (158)		
4	3.3% (75)		
5	0.6% (13)		
	**Opportunities per semester for** **participation**	**# of students** **participating per school each semester** **M (SD)**	**Frequency of participation per student each semester** **M (SD)**
	
**Public Commitment Activities**			
Event specialist	1 - 6	6.8 (3.8)	1.9 (0.9)
News reporter	1 - 11	4.6 (3.2)	2.6 (2.1)
Classroom assistant	1 - 26	9.2 (2.8)	7.6 (3.3)
Photojournalist	1 - 4	3.4 (2.3)	1.3 (0.5)
SGM	1	13.7 (4.6)	N/A

N = 2307 consented students from intervention schools only; SPC - student peer communicator; SGM - student-generated media; ***event specialist ***includes helping organize, facilitate and inform HEALTHY events such as cafeteria learning labs, taste tests, and assemblies; ***news reporters ***delivered PA announcements school-wide and in classrooms; ***classroom assistants ***helped with reading aloud and demonstrating activities in core classrooms and PE classes; ***photojournalists ***took pictures and designed photo collages; and ***SGM ***indicates participation in photo shoots when picture and testimonial statements were included on posters displayed throughout the school

### Public Commitment Activities: Student-Generated Media

As part of the communications campaign, identical centrally-produced posters, signage, and cafeteria-line messages were displayed at all intervention schools throughout the first year of the intervention. One year into the intervention, in the spring of 7^th ^grade, the communications approach changed to involve students in the process. This shift involved creation of "Student-Generated Media" (SGM) as the core of the communications approach. Study-wide poster and DVD templates consistent with each semester's intervention theme were provided to the local study sites' staff, which then conducted school-specific photo shoots and allowed students to provide photographs, artwork, audio clips, and video clips for use in the intervention's communications campaign. Accordingly, communications materials distributed throughout the latter stages of the intervention depicted study students' own experiences and highlighted their public commitment to the study and its healthy lifestyle goals. Because all SGM-created materials were posted for a minimum of several weeks in a given semester, participants' public commitment to the HEALTHY program was evident over a substantial portion of the semester even if the creation of a given poster required a relatively shorter time commitment. A more complete description of the development and implementation of this intervention element is provided elsewhere [34]. Participation in these activities was voluntary and required prior parental approval.

### Study Measures

Demographic, behavioral, and anthropometric data were collected at the study onset (beginning of 6^th ^grade) and conclusion (end of 8th grade). A comprehensive description of all study measures is included elsewhere [35]. For this report we also reviewed process evaluation data obtained throughout the study, including: qualitative student feedback obtained via structured interviews and focus groups; SPC tracking logs recording each time an SPC received training or participated in a study-related activity; and SGM logs identifying students whose pictures, art-work or video clips were used in study-related communications materials. These data were collected at each intervention school every semester. A complete description of process measures for the study was published previously [42].

### Analysis Plan

A total of 4603 students provided complete and valid primary outcome data in both 6^th ^and 8^th ^grade. Smaller student subsamples also provided complete and valid data for waist circumference, dietary intake, and fitness in 6^th ^and 8^th ^grades. Descriptive statistics with means (M) and standard deviations (SD) for continuous measures and percentages for categorical variables are presented. While the school was the unit of randomization and intervention, for this report we further divided the students in intervention schools into two groups: those who participated in public commitment activities (PC) and those who did not participate (NPC). Hence, for this report we examined differences between the three groups of PC, NPC and control. It should be noted that inclusion in the PC and NPC group was not determined by randomization but rather by self-selection. General linear mixed models (GLMM) were used to analyze differences between these groups or clusters [43,44] with the covariance structure appropriately adjusting for variability both between cluster and also within cluster [36,45]. This was accomplished by using the PROC MIXED procedure for continuous data and the PROC GLIMMIX procedure for categorical data incorporating a random effect for school at baseline and for group assignment within school at end-of-study into the model. Model selection was performed by including characteristic variables, where appropriate, into the model both alone and as an interaction with group assignment and any variables with a p-value greater than 0.10 in either instance were removed from the overall model. Models for demographic and anthropometric baseline variables were unadjusted, and models for anthropometric end-of-study variables were adjusted only for their baseline value (e.g. models for end of study BMI percentile contained the baseline BMI percentile as a covariate). Models for dietary intake and fitness variables were adjusted for baseline value at the end of study and for gender at both baseline and end-of-study (e.g., models for baseline fitness had gender as a covariate while the models for fitness at the end of study contained both gender and the baseline number of laps as covariates). Sexual maturation or pubertal stage was determined using the gender-specific pubertal development scale [46,47] from which Tanner score was determined [48]. When the resulting Tanner score was included in the exploratory models for fitness, it was found to contribute very little and its inclusion did not affect the overall fitness results. Hence, the final models were not adjusted for Tanner score. Due to skewness and presence of zeros, the dietary intake variables were transformed using the square root to ensure approximately normal distributions of the variables. When statistically significant group differences were found, pairwise comparisons were carried out to evaluate meaningful differences between categories. To account for multiple testing and protect the family-wise error rate, a modified sequentially rejective Bonferroni procedure was used to adjust p-values [49].

Consistent with the recently published report examining main outcomes of the study [37], the same outcomes were assessed in a high-risk subgroup of overweight or obese (BMI > 85th percentile) students at baseline; 50% of participating students met this criterion (Table 2).

**Table 2:** Baseline Measures by Participant Group

	**Overall (N = 4603)**				**Baseline BMI ≥ 85**^**th **^**percentile (N = 2292)**			
	**PC (N = 835)**	**NPC (N = 1472)**	**Control (N = 2296)**		**PC (N = 392)**	**NPC (N = 768)**	**Control (N = 1132)**	
	**Mean (SD) or %**	**Mean (SD) or %**	**Mean (SD) or %**	**p-value**	**Mean (SD) or %**	**Mean (SD) or %**	**Mean (SD) or %**	**p-value**
		
Student Characteristics								
Age in years, M (SD)	11.2 (0.5)	11.3 (0.5)	11.3 (0.5)	0.08	11.2 (0.5)	11.2 (0.5)	11.3 (0.6)	0.14
% male^1^	41.4%	50.7%	47.1%	< 0.001	47.2%	52.2%	50.4%	0.24
Race/Ethnicity				0.58				0.40
Hispanic	51.0%	57.0%	53.5%		53.8%	60.7%	56.7%	
Black	22.5%	19.1%	15.7%		19.9%	17.7%	16.2%	
White	18.6%	16.2%	21.6%		17.9%	14.3%	18.6%	
Other	7.9%	7.7%	9.2%		8.4%	7.3%	8.5%	
BMI Percentile, M (SD)	71.5 (28.1)	74.1 (27.5)	72.3 (28.6)	0.08	94.9 (4.1)	95.1 (4.0)	95.1 (4.1)	0.66
BMI ≥ 95^th ^Percentile	27.8%	31.4%	30.4%	0.20	59.2%	60.2%	61.7%	0.61
Waist Circumference ≥90^th ^Percentile	28.0%	30.3%	28.6%	0.47	59.3%	57.7%	57.7%	0.92
Family Characteristics								
Highest Household Education^3^				0.25				0.28
≤ HS Graduate	48.7%	53.6%	51.5%		49.1%	56.5%	54.9%	
≥ Some College	51.3%	46.4%	48.5%		51.0%	43.5%	45.1%	
Positive Reported Family History of Diabetes	14.5%	11.4%	13.4%	0.08	18.9%	14.2%	17.7%	0.07
Student Health Behaviors								
Fruits and Vegetables (servings/day), M (SD)^4^	2.6 (2.3)	2.7 (2.4)	2.8 (2.6)	0.64	2.6 (2.2)	2.6 (2.3)	2.8 (2.6)	0.37
Added Sugar Beverages (oz/day), M (SD)^4^	11.5 (14.4)	11.1 (13.2)	10.8 (12.4)	0.97	10.9 (13.4)	11.1 (13.4)	10.2 (11.9)	0.65
Fitness (# of laps), M (SD)^7,8^	22.2 (12.2)	20.4 (11.6)	21.4 (12.3)	0.0043	17.2 (9.8)	16.0 (8.5)	16.1 (8.3)	0.15

PC = group of intervention school students engaged in public commitment activities, NPC = group of intervention students not engaged in public commitment activities

^1 ^For overall sample, analyses of % male: PC vs. NPC, p = < 0.001; PC vs. Control, p = 0.02; NPC vs. Control, p = 0.09

^2 ^"Other" race/ethnicity not used in the analysis;

^3 ^N = 4471 for overall analyses and N = 2236 for BMI ≥ 85^th ^percentile subgroup due to missing data;

^4 ^N = 3908 for overall analyses and N = 1937 for BMI ≥ 85^th ^percentile subgroup due to missing data; ^5^adjusted for gender;

^6^Square root transformation used for analysis

^7 ^N = 4157 for overall analyses and N = 2069 for BMI ≥ 85^th ^percentile subgroup due to missing data;

^8 ^For overall sample, analyses of fitness: PC vs. NPC, p = 0.001; PC vs. Control, p = 1.00; NPC vs. Control, p = 0.377

As previously reported [36], the power calculation for this study was based on detecting change in the prevalence of overweight and obesity. As such the p-values reported within this paper are associated with exploratory, post-hoc analyses, represent findings associated with secondary outcomes, and are provided to help facilitate the interpretation of the data only with alpha set at .05. All analyses were performed using SAS version 9.2 (SAS Institute, Cary, NC).

## Results

### Baseline Characteristics

Table 2 compares the baseline characteristics of the three groups: consented students in the intervention schools who did and did not participate in public commitment activities (i.e., PC vs. NPC) and consented students in the control schools. Comparisons are presented for the entire sample and for the subgroup of students who were overweight or obese at baseline. Few differences emerged at baseline between the groups. For the overall sample, a significantly smaller proportion of males were in the PC group than was anticipated given the number of males in the sample (41.4% versus 50.7% in the NPC group and 47.1% in the control group, p < 0.001). Hence, gender was added as a covariate into the outcome analyses for student health behaviors (see Tables 2 and 3). However, because anthropometric variables (BMI percentile, waist circumference ≥ 90^th ^percentile) already adjust for gender, it was not included in the outcome analyses of these variables to prevent over-parameterization. The PC group scored higher on the fitness measure, completing more laps (M = 22.2 [SD = 12.2]) compared to the NPC group (M = 20.4 [SD = 11.6], p = .001). Although statistically significant, the modest difference in number of laps completed between the groups suggests a non-meaningful fitness difference at study onset [50,51]. Baseline fitness was added into the models as a covariate with only slight changes in overall significance detected. For this reason fitness was not retained in the final models. There were no significant differences on nutritional variables (fruit/vegetable and added sugar beverage consumption) between either of the intervention groups (PC, NPC) and the control group. Finally, there were no differences in baseline characteristics or health behaviors between the PC, NPC and control groups when restricting the sample to those who were overweight or obese at baseline.

**Table 3:** Anthropometric and Health Behavior Outcomes by Participant Group

	**Overall (N = 4603)**				**Baseline BMI ≥ 85**^**th **^**percentile (N = 2292)**			
	**PC (N = 835)**	**NPC (N = 1472)**	**Control (N = 2296)**		**PC (N = 392)**	**NPC (N = 768)**	**Control (N = 1132)**	
	**Mean (SD) or %**	**Mean (SD) or %**	**Mean (SD) or %**	**p-value**	**Mean (SD) or %**	**Mean (SD) or %**	**Mean (SD) or %**	**p-value**
		
BMI Percentile, M (SD)	70.9 (26.9)	73.2 (26.3)	72.6 (26.7)	0.20^1^	91.3 (9.7)	91.7 (9.9)	92.2 (8.9)	0.17^1^
BMI ≥ 95^th ^Percentile	21.4%	26.4%	26.6%	0.02^1^	44.6%	50.1%	53.2%	0.01^1^
Waist Circumference ≥90^th ^Percentile	19.7%	22.2%	22.7%	0.07^1^	40.4%	41.8%	45.1%	0.05^1^
Student Health Behaviors								
Fruits and Vegetables (servings/day), M (SD)^4^	2.4 (2.0)	2.4 (2.1)	2.3 (2.0)	0.23^5^	2.4 (2.2)	2.3 (2.0)	2.3 (2.1)	0.62^5^
Added Sugar Beverages (oz/day), M (SD)^4^	12.5 (12.3)	13.5 (13.9)	14.3 (15.2)	0.31^5^	10.9 (11.0)	13.0 (13.3)	12.9 (14.2)	0.20^5^
Fitness (# of laps), M (SD)^7,8^	28.3 (17.7)	26.6 (16.5)	27.6 (17.3)	0.42^5^	25.3 (16.2)	23.1 (14.8)	22.9 (14.9)	0.35^5^

PC = group of intervention school students engaged in public commitment activities, NPC = group of intervention students not engaged in public commitment activities

^1^Adjusted for baseline value

^2 ^For overall sample, analyses of BMI ≥ 95^th ^percentile: PC vs. NPC, p = .05; PC vs. Control, p = 0.02; NPC vs. Control, p = 0.37. For high risk (baseline BMI ≥ 85^th ^percentile) analyses of BMI ≥ 95^th ^percentile: PC vs. NPC, p = .05; PC vs. Control, p = 0.01; NPC vs. Control, p = .27

^3 ^N = 4587 for overall analyses and N = 2282 for BMI ≥ 85^th ^percentile subgroup due to missing data

^4 ^N = 3908 for overall analyses and N = 1937 for BMI ≥ 85^th ^percentile subgroup due to missing data;

^5 ^Adjusted for baseline value and gender; ^6^square root transformation used for analysis;

^7^N = 4157 for overall analyses and N = 2069 for BMI ≥ 85^th ^percentile subgroup due to missing data

### Public Commitment Activity

Table 1 summarizes student participation in public commitment activities. Thirty six percent of consented students in intervention schools participated in some form of public commitment activity (the PC group), with approximately equal numbers participating in SPC activities (13.8%) and SGM activities (13.5%) and a smaller proportion participating in both (8.9%). In addition, Table 1 suggests broad involvement across the available activities. Examination of the interview and focus group data obtained through process evaluation confirms that the activities we classified as evidence of public commitment were, in fact, noticed and remembered by students in the intervention schools. In interviews conducted each semester, approximately 90% of students consistently reported knowing one or more SPC at their school, and more than 60% of students suggested that the SPCs were instrumental in helping them make healthier lifestyle choices. Moreover, the interviews indicated that the student-generated media were considerably more salient than the centrally-produced materials featured in the earlier semesters. Across the two semesters spanning the transition from centrally-produced to student-generated media, there was a roughly 50% increase (48% to 74%) in the proportion of interviewed students who, unprompted, reported noticing study posters throughout the schools.

### Outcome Analyses

Table 3 presents anthropometric and health behavior outcomes at the end of the study, and compares the PC group, the NPC group, and the control group. Within the overall study, including all 4603 consented students, a lower proportion of the PC group was obese at the end of the study compared to the control group (21.4% vs. 26.6%; p = .02). In contrast, there was no difference in obesity rates between the NPC group and the control group (26.4% vs. 26.6%; p = .37). In other words, the data suggest the intervention had no effect on the NPC group, but reduced obesity prevalence within the PC group. At the end of the study, the difference in obesity prevalence between the PC group (21.4%) and the NPC group (26.4%) approached significance (p = .05). Each outcome variable was adjusted for baseline values.

The differences in obesity prevalence were even greater among the subgroup of students who were either overweight or obese at baseline. In this subgroup analysis, the PC group had lower prevalence of obesity (44.6%) at the end of study compared to the control group (53.2%; p = 0.01). There was no difference in this subgroup analysis between obesity rates for the NPC group (50.1%) and the control group (53.2%; p = 0.27). As with the analysis of the total study sample, when the analysis was limited to the students who were already overweight or obese at baseline, the intervention reduced the prevalence of obesity within the PC group but not within the NPC group. Finally, in the subgroup analysis, the difference in obesity prevalence at the end of study between the PC group (44.6%) and the NPC group (50.1%) approached significance (p = .05). Again, these analyses were adjusted for the corresponding baseline value.

We found an overall significant effect of group on waist circumference at or above the 90^th ^percentile. However, adjusted pairwise comparison analyses did not suggest statistically significant differences between the groups. Analogous analyses of student health behaviors revealed no overall effect of group on fitness or nutrition measures; this lack of effect extended to both intervention groups, regardless of their participation in public commitment activities.

### Dose Analyses

Finally, to examine the relationship between greater involvement in public commitment activities and improved anthropometric and health behavior outcomes, we totaled the number of semesters that each intervention student participated in any public commitment activities. Students could participate in a maximum of five semesters of such activities (spring of 6^th ^grade through spring of 8^th ^grade). None of the analyses suggested that "dose" of public commitment activities significantly impacted study outcomes.

## Discussion

We examined whether making a public commitment to program goals in a school-based intervention to reduce obesity predicted improved study outcomes. We found that the intervention had a significant effect on the prevalence of obesity within the group that participated in public commitment activities, although there was no intervention effect within the group that did not participate in public commitment activities. Consistent with the main outcome findings from the overall HEALTHY study [37], the effect was magnified among the subgroup of students who were overweight at baseline. Within the intervention schools, the group not participating in public commitment activities were nearly as likely to be obese (≥ 95^th ^BMI percentile) as the control group. These results indicate that participating in public commitment activities during the HEALTHY study may have potentiated changes promoted by the behavioral, nutrition, and physical activity intervention components.

Importantly, there were no substantive differences in measured baseline characteristics between the group that participated in public commitment activities and the group that did not, suggesting that these results were not an artifact of differences in measured variables at study onset. This finding was not altogether expected, as we anticipated that students who had close family members with diabetes or who were healthier at onset (e.g., not overweight/obese, better reported dietary and physical activity patterns) might have been more likely to participate in study activities and elect to join the PC group. Interestingly, it appeared that the act of participation--rather than amount of participation--in public commitment activities was the critical factor in affecting outcomes. Students with multiple semesters, compared to a single semester, of public commitment participation demonstrated no better outcomes.

Review of our process data supported our assumption that participation in SPC and SGM activities demonstrated a public commitment to program goals. The communications campaign was designed to shift social norms and create a positive image of the intervention as a whole; interview testimony showed clear recognition throughout the student body regarding SPCs' visibility as well as evidence of their effectiveness as agents of influence. Moreover, the considerable increase in awareness of study posters following the introduction of student-generated media highlighted the salience of these images. Nonetheless, we wish to emphasize the importance of the core intervention elements (nutrition and physical activity offerings as well as behavioral change campaigns) in building the foundation for such changes. We are skeptical that the implementation of public commitment activities such as those described here would contribute to a lower prevalence of obesity in the absence of these core elements.

Our findings are consistent with others who have reported that peer leadership participation results in better health-related outcomes than participation in the core intervention only [33,52-54]; although, to our knowledge, none of these previous studies included a student-generated media component, nor did they examine findings within the framework of the influence of public commitment activities. Indeed, our finding that activity dose was not related to outcome suggested that it was the commitment per se rather than the potential added exposure to the intervention that was associated with the observed lower prevalence of obesity.

With the greater reduction in obesity prevalence within the group participating in public commitment activities, we expected to see some changes in the hypothesized mediators; namely, improved fitness and dietary intake. No such differences were detected. Our observations may have been restricted by the limited sensitivity of our dietary intake and physical activity assessment measures (Block Kids FFQ; 20-meter shuttle run). More sensitive measures might have revealed mechanisms that affected obesity prevalence. Future studies may benefit from more sensitive assessments in evaluating proposed mediators of change.

Some caution should be exercised in generalizing from the present study, as the findings are exploratory and post-hoc. As such we characterize the analyses presented here as "hypothesis generating" suggesting that an important next step is replicating the study using a fully powered "hypothesis testing" design. In addition, our analysis did not discriminate between the impact of the different modes of public commitment on study outcomes. Future studies may want to examine the impact of the various types of public commitment activities separately (e.g., peer leadership and student-generated media). Further, we can not rule out that differences in outcomes may have been due to unmeasured factors (e.g., prosocial behavior that motivated student participation in these public commitment activities as well as uptake of HEALTHY behavioral targets). Finally, an inherent limitation of the research is the non-randomized nature of the "public commitment" assignment. While conceivably a study design (however cost-prohibitive) could randomly assign students to public-commitment activities, we suspect that the voluntary nature of this undertaking is important and perhaps inherent to its success.

As concern about youth obesity continues to mount, widespread policy changes supporting behavior-change initiatives and nutritional and physical activity offerings in schools may have enhanced impact with the inclusion of the incremental benefit of public commitment activities. Sustaining public commitment activities in everyday school settings (without the support of a clinical trial) would require a teacher or school administrator to organize the opportunities for such activities yet the program itself requires little investment. This approach is also likely to be implementable across a broad range of schools and communities as the largely student led nature of such activities ensures that youth experiences and sensibilities in a given setting are accurately reflected in a manner that would not be possible with a more centrally based communications approach. The next step for such research may involve randomizing schools to receive the additional element of public commitment activities on top of publicly funded and mandated obesity prevention programs. We found that working within a middle school environment, there were often administrative, pragmatic and/or safety concerns that limited our ability to use a broader set of technologies and social media to further student options, particularly ones that would be acceptable across all 21 of the intervention schools. This experience suggests that single-site studies may be able to employ more innovative means to further student-generated opportunities for program involvement thereby perhaps potentiating the effect of such public commitment activities on health outcomes.

## Conclusions

As concern about youth obesity continues to mount, there is increasing consideration of widespread policy changes to support improved nutritional and enhanced physical activity offerings for middle school students - a development period of heightened risk for the development of obesity and consequently an important opportunity for prevention. Our research suggests that a critical element in the success of such programs may be to involve students as spokespeople for the program. Making such a public commitment to healthy lifestyle program targets (improved nutrition and enhanced physical activity) may potentiate healthy behavior changes among such students and provide a model for their peers.

## Competing interests

The authors declare that they have no competing interests.

## Authors' contributions

LLD led the development and revision of the manuscript. KLD preformed the analyses and led the interpretation of the data. LLD, MS, and KLD played a major role in the revision of the manuscript for intellectual content. LLD, MS, KLD, EGF, DDS, ELM, MW, AEH, SS, AJ were responsible for conception and design of the communications component of the HEALTHY study. All authors participated in the implementation of the study, interpretation of data, and development and revision of the manuscript. All authors read and approved the final manuscript.

## Pre-publication history

The pre-publication history for this paper can be accessed here:

http://www.biomedcentral.com/1471-2458/11/711/prepub
